# Web-based standardized patient simulation for taking anamnesis: an approach in nursing education during the pandemic

**DOI:** 10.1186/s12912-023-01486-4

**Published:** 2023-09-20

**Authors:** Ayse Demiray, Nagihan Ilaslan, Selin Keskin Kızıltepe, Aysegül Acıl

**Affiliations:** Fundamentals of Nursing, Nursing Department, Faculty of Health Sciences, Duzce University, Duzce, Turkey

**Keywords:** COVID-19 pandemic, Nursing education, Nursing students, Simulation, Standardized patients, Web-based intervention

## Abstract

**Background:**

To address the challenges in nursing education brought about by the pandemic, this study aimed to evaluate the use of a web-based standardized patient practice in the development of nursing students’ anamnesis taking skills and their views about its application.

**Method:**

We conducted a descriptive intervention study with 39 s-year nursing students. The students completed anamnesis using the standardized patient practice in line with a scenario with real standardized patients in a web-based environment with audio and video.

**Results:**

The total scores of students’ anamnesis skills were low. The agreement between the total scores, scores obtained from the health patterns, and each item in the control list was statistically significant (p < 0.05).

**Conclusion:**

Web-based standardized patient practice is an alternative for clinical practice facilitating the gaining of competencies in making holistic nursing diagnoses under conditions that limit face-to-face interactions, such as pandemics.

## Introduction

Nursing care should be conducted effectively to protect and improve the health of individuals, families, and society [1]. The importance of the professional nursing workforce, with adequate and effective nursing training, is better understood today in achieving proper nursing care. Particularly considering nursing care during the coronavirus disease 2019 (COVID-19) pandemic, the year 2020 has been declared the year of nurses and midwives by the World Health Organization [2–4]. However, nursing education, wherein the professional nurse workforce is trained, varies globally [5]. This variability has further increased with the challenges brought by the COVID-19 pandemic in nursing education. Particularly, it has been emphasized that the main challenges of the pandemic are related to clinical nursing education [6]. Clinical and typical face-to-face simulation practices could not be performed in many countries because of the COVID-19 pandemic; therefore, virtual simulation techniques were frequently preferred and employed instead [7, 8].

Virtual simulations in nursing education include interactive patient scenarios, software simulations, and virtual standardized patients [9, 10]. The increase in the use of virtual patients using specific software has drawn considerable attention [7, 11–13]. However, Shorey and Debby (2020) reported that virtual patient software requires high technological investment, creates a low sense of reality, and is more effective only in the acquisition of cognitive competencies (such as, systematic thinking, remembering the order of steps of activities, etc.), unlike psychomotor skills. Additionally, it has been reported that virtual patient software has high costs, which restrict its use [12, 14]. Hence, it is difficult for educators who do not have financial resources, technical equipment, or experience to access and use virtual simulation software. Therefore, educational institutions and educators require innovative alternatives that can be used to teach some of the clinical practice skills without virtual patient software [15]. One of these skills is the ability to take anamnesis as the nursing process begins with data collection. The interview method is a data collection method which can be used systematically by following a standard procedure. One of the most widely used standard procedures worldwide is Gordon’s Functional Health Patterns Model [16].

In response to this need, this study focused on designing an innovative web-based standardized patient simulation experience (with real standardized patients on a free-to-use web-based platform via an internet connection) as an alternative for clinical practice for taking anamnesis using the interview method at a state university during the COVID-19 pandemic. Compared to the expensive virtual simulation software, this method required money only for the working hours of the real standardized patients [14]. This study aimed to examine the role of a web-based standardized patient simulation in nursing students’ learning of the ability to take anamnesis and to determine their perceptions of this experience. The study makes an important contribution to the literature by revealing the usability of an alternative simulation practice that can be quickly integrated into the education process in times of crises such as the pandemic, which adversely affects nursing education.

## Theoretical framework

Standardized patients are defined as healthy individuals or real patients who have been trained to behave consistently in the patient’s role within the framework of the scenario created [17]. The Functional Health Patterns Model developed by Marjory Gordon in 1987 guided the use of anamnesis in standardized patients in this study. This model was selected because it is routinely used for nursing assessments performed by students in their clinical practice at the university where the study was conducted. This model was used to systematically collect and interpret patient data and establish comprehensive nursing data. In the model, 11 health patterns (Health Perception-Health Maintenance (experiencing in thoughts about health of change), Nutrition‐Metabolic (experiencing anorexia, nausea, and vomiting), Elimination (experiencing constipation, diarrhea, stool incontinence), Activity‐Exercise (activity-exercise levels, experiencing difficulty in eating-drinking, going to toilet, bathing, dressing and moving), Sleep‐Rest (experiencing sleeping problems, e.g. sleepingless than normal, sleeping more than normal, difficulty falling a sleep, waking up in midnight, not waking up rested in the morning), Coping‐Stress, Self‐Concept‐Self‐Perception (effective practices to reduce stressful situations), Cognitive‐Perceptual (experiencing pain and its severity), Role‐Relationship (experiencing difficulty in fulfilling roles and responsibilities and experiencing difficulty in maintaining social relations because of the disease), Sexuality‐Reproductive, and Value‐Belief (changes in sexual life because of the disease)) are defined, with the aim of evaluating the individuals as a whole and providing holistic nursing care [18].

## Method

### Study design

This was a mixed methods study conducted to assess nursing students’ baseline ability to take anamnesis through web-based standardized patient simulation [19].

### Participants and setting

The study sample included 40 s-year nursing students taking the Physical Assessment Course at a state university. The sample was calculated based on the power analysis (95% confidence interval) (0.5 effect size, theoretical power = 80%). The students were selected by one of the researchers using simple random sampling method via simple random numbers Table [19]. Because one student did not want to participate in the study, it was completed with 39 students who voluntarily participated in the web-based standardized patient simulation.

### Designing the scenario and web-based simulation

The scenario related to chest disease (an adult patient diagnosed with asthma) was developed by researchers who had training in scenario writing. The scenario was structured to cover all of the dimensions of the Gordon’s model. Feedback about the scenario was taken from two nursing faculty who had simulation education experience and two registered nurses who worked with adult patients at a chest disease clinic.

The web-based simulation design followed the International Nursing Association for Clinical Simulation and Learning Standards of Best Practice [20]. In this process, all the simulation stages were completed: pre-briefing (theoretical notes were sent to students about history taking, web links for simulation were sent before practice, and one pre-session was conducted to anticipate web-based connection problems), briefing (online information about the scenario was provided), practiced scenario, and debriefing.

#### Preparation of the standardized patients

The study was conducted with four standardized patients (who were healthy and had been trained to behave consistently in the patient’s role) registered at the Standardized Patients Program of a state university; all the standardized patients were educated on the Association of Standardized Patient Educators Standards of Best Practice [21], and had standardized patient experience of nearly ten years. One week before the practice, the guide for the scenario prepared by the researchers was sent to the standardized patients via an e-mail. The guide included the questions for each sub-dimension that the students were expected to ask and their answers within the scope of the scenario designed based on Gordon’s model. A pre-practice was performed with all standardized patients a day before the simulation practice to ensure role similarity based on the scenario.

### Data collection instruments

The descriptive characteristics of the students, including age, sex, and achievement levels, were collected using a questionnaire consisting of six items. For determining the students’ ability to take anamnesis, a control list related to taking anamnesis, which was constructed by researchers in line with Gordon’s model, was used [16]. The control list consists of 49 questions with a two-point degree (assessed [one point] or non-assessed [zero points]) for each of the 11 health patterns; a maximum of 49 points can be obtained. The content validity of the control list was established by three nursing faculty members outside the research team. In addition, students’ web-based simulation perceptions were investigated using a self-debriefing form that was prepared by the researchers based on literature in accordance with the Promoting Excellence and Reflective Learning in Simulation (PEARLS) framework [11, 19, 22]. In the self-debriefing form, the students were asked about their thoughts on the practice, to evaluate themselves, state the positive and negative aspects of the practice, and their suggestions.

### Data collection

Data were collected between December 1 and 8, 2020. Standardized patients, students, and researchers simultaneously participated in the simulated scenario online using a mobile phone or computer with a microphone and camera. The practice hours of each student were predetermined based on the students’ extracurricular times. Each of the standardized patients participated in the patient role for ten students, as long practice hours can decrease standardized patients’ role consistency. The researchers evaluated the students’ ability to take anamnesis by observing and listening through the computer screen during the simulated scenario, using the control list constructed in line with Gordon’s model. Web-based standardized patient practices were completed within an average of 20 min. Immediately after the practice, students performed self-debriefing using a self-debriefing form consisting of seven open-ended questions. The form was delivered to students via e-mail. Students wrote their answers to open-ended questions in the spaces below the questions and sent the completed forms to the researchers via e-mail.

### Data analysis

Descriptive statistics (mean, standard deviation, percentages) were used to determine students’ demographic data and ability to take anamnesis using IBM SPSS Statistics software (version 22.0) for Windows. Since the students’ ability to take anamnesis was evaluated independently by two researchers, the inter-observer compliance among the total scores (Intra class correlation coefficient [ICC]) and each item of the control list (Kappa analysis) that the researchers gave to students was analyzed. The researchers analyzed the students’ perceptions of the web-based standardized patient experience using content analysis.

### Ethical considerations

The researchers received ethical approval (approval year/number: 2020/183) from the ethics board of the university where the study was conducted. The aim of the study was explained to both standardized patients and students, and all participants provided written informed consent to participate in the study. The web link for the simulation experience was kept open only during the simulation sessions, and then access to the link was blocked by the researcher who created the online web sessions.

## Results

The average age of the participants was 19.50 ± 0.73; 71.1% (n = 27) were female, and 84.2% (n = 32) were Anatolian (public) high school graduates. The academic achievement average of 73.7% (n = 28) was found to be between 2.50 and 3.00. Additionally, the students had not participated in any simulation training prior to our session.

The results revealed that observers consistently evaluated the characteristics of prosthesis use, (special) diet, frequency of defecation, date of last defecation, defecation problems, breast or testis examination status, regular health checks, smear test status, last test date, sexual problems, and menstrual cycle features. Complete agreement was observed between them. There was no inter-observer agreement regarding supportive treatments or over-the-counter drugs. Inter-observer consistency was found for 93.88% of the 49 items (Table 1).

**Table 1:** Inter-observer consistency reliability for anamnesis skill

Evaluation criteria		Kappa	p
Current illness history	Complaints (pain, fatigue, nausea, etc.), anatomical region of the complaint	0.537	0.000
	The time	**0.133**	**0.326**
	Incidence frequency	0.855	0.000
	Severity	0.682	0.000
	Increasing and decreasing factors	0.650	0.000
Past medical history	Past consultation with the hospital with the same complaint within 6 months	0.532	0.000
	Previous hospitalization experience and previous surgical operation experience	0.649	0.000
	Drugs used, doses, frequency of use	0.790	0.000
	Supportive therapies used	**0.131**	**0.385**
	Presence of allergy	0.934	0.000
	Using a prosthesis	**1.000**	**0.000**
	Supportive tool use	0.479	0.001
Family/genealogical background information	Illness in the family, the presence of hereditary disease	0.934	0.000
Information about social/business life	Occupation, health insurance	0.821	0.000
	People who receive support in treatment and care	0.894	0.000
	City/region of residence	0.917	0.000
Nutrition/metabolic status	Diet	**1.000**	**0.000**
	Special diet presence	**1.000**	**0.000**
	Normal eating habits	0.650	0.000
	Problems with nutrition	0.874	0.000
	Height and weight	0.947	0.000
	Weight loss	0.843	0.000
Excretion	Defecation (solid or liquid)	0.787	0.000
	Frequency of defecation, date of last defecation	**1.000**	**0.000**
	Presence of defecation problem	**1.000**	**0.000**
	Form of diuresis	0.723	0.000
	Diuresis problems	**1.000**	**0.000**
Sleep/rest	Sleep duration/hours	0.767	0.000
	Pre-sleep habits/routines	0.214	0.033
	Feeling rested after sleep	0.630	0.000
	Daytime sleep	**1.000**	**0.000**
	Free time activities	0.917	0.000
Health perception and promotion	Alcohol use	0.917	0.000
	Smoking	0.907	0.000
	Exercising	0.642	0.000
	Non-prescription drug use	**0.036**	**0.811**
	Illicit substance use	0.655	0.000
	The state of performing self-care practices	0.723	0.000
	Having a breast/testicular examination	**1.000**	**0.000**
	Regular health checkups	**1.000**	**0.000**
	Smear test/last test date	**1.000**	**0.000**
Coping/stress tolerance	Expectation of treatment and care	0.374	0.003
	Concerns about the disease	0.802	0.000
	Concerns about hospitalization	0.770	0.000
	Common coping methods	0.655	0.000
Value and belief	Practices that he/she wants/does not want to be done in line with his/her belief	0.874	0.000
Sexuality/reproduction	Sexual problems	**1.000**	**0.000**
	Menstruation cycle features	**1.000**	**0.000**
	Presence of pregnancy,curettage,abortus	0.655	0.000

*Note.p < 0.001*

Regarding the results of the consistency of the observer score averages for the patterns related to the ability of taking anamnesis, the agreement between the mean scores of Observers 1 and 2 was found to be statistically significant for all parameters (p˂0.001) (Table 2).

**Table 2:** In-class evaluation coefficients for anamnesis skills

Evaluated dimensions	Observer 1 Mean ± SD	Observer 2 Mean ± SD	*ICC*	p
Current illness history	2.11 ± 1.23	2.16 ± 1.26	0.894	0.000
Past medical history	3.16 ± 1.50	2.95 ± 1.33	0.861	0.000
Family/genealogical background information	0.71 ± 0.46	0.74 ± 0.45	0.967	0.000
Information about social/business life	1.45 ± 0.89	1.39 ± 0.89	0.947	0.000
Nutrition/metabolic status	1.53 ± 1.53	1.44 ± 1.41	0.979	0.000
Excretion	0.47 ± 1.11	0.39 ± 0.97	0.970	0.000
Sleep/rest	0.97 ± 1.28	1.08 ± 1.38	0.962	0.000
Health perception and promotion	1.87 ± 0.99	1.95 ± 1.14	0.918	0.000
Coping/stress tolerance	0.42 ± 0.83	0.36 ± 0.75	0.932	0.000
Value and belief	0.13 ± 0.34	0.11 ± 0.31	0.935	0.000
Sexuality/reproduction	0.08 ± 0.27	0.05 ± 0.23	0.883	0.000
Total Points	12.89 ± 6.56	12.63 ± 6.66	0.979	0.000

*Note.*ICC: Intraclass correlation coefficient, SD:Standard deviation,p < 0.001

The statements regarding the students’ perceptions of the web-based standardized patient practice were grouped into five categories during the analysis. Table 3 shows all of these categories, including the short excerpts of the expressions conveyed by the students.

**Table 3:** Students’ views on web-based standardized patient practice

Categories	Student expressions
1. Feelings during practice	*“I felt very excited. On the other hand, I was happy. I felt like I had really become a nurse…”*(S10) *“…Since we didn’t get an internship at the hospital, I felt deficient in myself. That’s why it feels so good to have such an opportunity…”*(S13) *“…It went well for me, even if it was exciting.” (*S2)
2. Students’ self-assessment according to their application skills	“*…Since I took anamnesis for the first time, I also had some shortcomings and inexperience… the value of these practices will increase for us,.especially if we are not able to be in the classes and in the hospital due to this pandemic.”*(S28) “*…I think it will be more productive when you are with the patient in the virtual environment. Thanks to this application, I saw my mistakes and understood that I had to overcome my excitement….*”(S16) *“I realized that I needed to develop the skill of asking questions to the patient…”*(S19)
3. Positive aspects of the application	“*…As a result of the questions I asked, I understood the patient’s answers and qualities, albeit partially, with his/her changing facial expressions…”*(S16) *“…It allows us to communicate with the patient since we could not go to the clinical practice. We can develop relations through the dialogue that will occur…”*(S18)
4. Negative aspects of the application	*“The negative side is that we only see what the patient shows us.…”*(S19) *“We cannot establish full face-to-face communication with the patient and cannot have an effective conversation. We are unable to perform the physical examination…”*(S21)
5. Suggestions for the application	“*Nursing students can improve themselves during the pandemic period by using such applications at certain intervals. …”*(S16) “*…For the implementation of the application, the creation of the necessary infrastructure that allows interviews through web-based, hospital, and even patient-specific channels will make a significant contribution to the applicability of the program.”*(S31) “*…Our teacher can organize a live interview about our shortcomings or make a collective criticism about his/her comments after using the application…”*(S23)

*Note.*S: Student

## Discussion

The total scores of the participants were considerably lower for the anamnesis skills, and the observers had a statistically significant agreement regarding the diagnosed health patterns. These results can be due to students continuing their nursing education in a completely web-based environment for approximately a year, and being unable to partake in any laboratory or clinical practice because of the COVID-19 pandemic. Thus, although web-based teaching methods during the pandemic period can provide students with theoretical knowledge, the ability to diagnose with a holistic perspective may still be underdeveloped as students cannot be taught how to apply this knowledge. When the results of a study conducted by the Turkish Association for Education in Nursing [23] were examined, it was found that in web-based education, students did not feel competent in learning. Additionally, 59.6% of the participants thought that nursing education could not be provided web based [23]. Similarly, in a report published by the International Council of Nurses (2021), 73% of national nursing associations registered with them stated that the COVID-19 pandemic had caused a major disruption in nursing education. Furthermore, 46% stated that clinical practice that was postponed or canceled caused a major deterioration in nursing education. They reported that this situation negatively affected the quality of nursing education. These findings were consistent with our results.

When students’ thoughts on the practice were examined, notably, the lack of clinical practice experience was reported as the main cause for them in feeling inadequate while taking anamnesis. Simultaneously, it is understood that the students found the web-based standardized patient practice useful for their development, as they could experience the feeling of being able to interact with the patient despite not being in the same environment. In Jimenez-Rodriguez and Arrogante’s (2020) study, in which counseling was performed with standardized patients through a web-based platform, the results revealed that the students found the application to be highly practical during the pandemic period and their satisfaction levels were high. This study supports our results. Notably, similar results have been obtained in different studies on this subject [19, 24]. In particular, performing applications with educated and experienced standardized patients provides opportunities for students to experience the feeling of patient interaction. Web-based applications with standardized patients are listed among the practical solutions that can be used in education during emergencies that may limit face-to-face interactions, such as pandemics [24]. In addition, the virtual standardized patient software is costly, and using real and trained standardized patients via web-based platforms can be more cost- and time-efficient for the educational institutions [13, 25, 26].

The teaching process in nursing education has experienced challenges worldwide [27–29]. However, since the level of inclusion of innovative teaching methods by educational institutions in the process differs depending on financial and workforce variables, there are concerns that the standardization and quality of nursing education [5, 30–33]. For this reason, the need for active teaching methods that can be used in web-based environments, such as simulations, is emphasized so that students can gain the expected competencies [34]. The results of our study suggest that real standardized patients can be used effectively via web-based platforms for training nursing students without any spatial and financial restrictions during acute crisis periods, such as pandemics. In addition to crisis periods, such simulations can be used actively in nursing education. For example, educational institutions that do not have a standardized patient program can cooperate with other institutions that have this program and integrate web-based standardized patient simulations into their education programs without time and cost limitations.

## Conclusions

The web-based standardized patient simulation is an innovative practice that can be used in the acquisition of holistic nursing diagnoses skills as an alternative to clinical practices that students cannot perform because of pandemic limitations. Although students’ level of satisfaction with the practice was high, the average scores for taking anamnesis skills were low. The inadequacy in skill levels, clinical applications that could not be realized during the pandemic period, and theoretical content realized with limited techniques reveal the need to include active and innovative teaching techniques. A web-based standardized patient practice, which removes space limitations without increasing the financial burden of educational institutions and provides the opportunity to interact with real individuals, serves to meet this need.

These web-based standardized patient practices can be used for some student practices planned in clinical settings during the ongoing pandemic. Therefore, the risk of transmission to students and healthcare professionals can be avoided by reducing the burden of clinical environments that maintain patient care in challenging conditions.

As web-based standardized patient practices eliminate time and space limitations, they can improve cooperation between students and educators in different institutions when the pandemic ends. This can serve to increase the standardization and quality of nursing education by increasing the chance of experiencing similar practices for students studying in institutions with financial and workforce limitations.

## Data Availability

The datasets used and/or analysed during the current study available from the corresponding author on reasonable request.
